# Conditional Values in Quantum Mechanics

**DOI:** 10.3390/e26100838

**Published:** 2024-09-30

**Authors:** Leon Cohen

**Affiliations:** Hunter College and Graduate Center, City University of New York, New York, NY 10065, USA; leon.cohen@hunter.cuny.edu

**Keywords:** conditional values, local values, Ehrenfest theorem, quantum mechanics, Bohm theory

## Abstract

We consider the local value of an operator for a given position or momentum and, more generally on the value of another arbitrary observable. We develop a general approach that is based on breaking up Aψ(x) as $\frac{A\psi (x)}{\psi (x)}={\left(\frac{A\psi (x)}{\psi (x)}\right)}_{R}\;+\;i{\left(\frac{A\psi (x)}{\psi (x)}\right)}_{I}$ where A is the operator whose local value we seek and ψ(x) is the position wave function. We show that the real part is related to the conditional value for a given position and the imaginary part is related to the standard deviation of the conditional value. We show that the uncertainty of an operator can be expressed in two parts that depend on the real and imaginary parts. In the case of the position representation, the expression for the uncertainty of an operator shows that there are two fundamental contributions, one due to the amplitude of the wave function and the other due to the phase. We obtain the equation of motion for the conditional values, and in particular, we generalize the Ehrenfest theorem by deriving a local version of the theorem. We give a number of examples, including the local value of momentum, kinetic energy, and Hamiltonian. We also discuss other approaches for obtaining a conditional value in quantum mechanics including using quasi-probability distributions and the characteristic function approach, among others.

## 1. Introduction

The concept of conditional values, sometimes called local values, is fundamental in any classical probability theory that involves multiple variables. For example, suppose we have a nonuniform gas characterized by a joint probability distribution of position and velocity. One can ask for the average velocity at a given position or the average kinetic energy at a given position. In quantum mechanics, the concept of local value runs into a fundamental difficulty in that, in general, two physical quantities, represented by operators, do not commute. Nonetheless, the concept of local values has arisen in numerous areas and has been used with considerable profit. Undoubtedly, the most well-known example of a local quantity is quantum mechanical current [1,2,3,4]. Also, the concept of local kinetic energy has been used in many studies regarding atoms in molecules [5,6,7,8,9,10]. It has also been used to develop quantum kinetic equations [11,12].

We shall discuss various approaches that have been developed and present a unified approach by developing a classical and quantum expression for the standard deviation of a physical quantity and comparing the two. Also, the issues we address here are analogous to those in the field known as time-frequency analysis [13,14,15,16,17,18,19,20,21]. In particular, for example, instantaneous frequency is mathematically analogous to quantum mechanical current. Much work has been conducted in that field, which is germane to the topics considered here.

### Notation

Operators are denoted by boldface letters and will be assumed to be Hermitian. All integrals go from −∞ to ∞ unless otherwise noted. For classical quantities, global expectation values will generally be denoted by $\langle a\rangle$ and conditional expectation by ${\langle a\rangle }_{x}$, meaning the conditional expectation value of *a* for a given *x*. Similarly, for the global standard deviation, we use $\sigma_{a},$ and for the conditional standard deviation, we use $\sigma_{a|x}.$

For the case of operators, the global expectation value, as usually defined, is denoted by $\langle A\rangle$ and the conditional expectation, which will be developed in this paper, by ${\langle A\rangle }_{x}$. The standard deviation of an operator, as usually defined in quantum mechanics, is denoted by ΔA, defined in the usual way:

(1) $${\left(\Delta A\right)}^{2}=\langle A^{2}\rangle -{\langle A\rangle }^{2}$$

For the conditional standard deviation, we use the notation ${\left(\Delta A\right)}_{x}$.

Commutators and anticommutators are denoted by the usual notation:

(2) [A,B]=AB−BA

and

(3) $${\left[A,B\right]}_{+}=\mathrm{AB}+\mathrm{BA}$$

respectively.

## 2. Local Values in Classical Physics

In this section, we develop the general ideas of conditional values and condition probability densities in standard probability theory. To keep things concrete, we develop the concepts for the case of an arbitrary variable, a, and position, *x*. We take the joint probability distribution to be P(x,a), of position *x*, and random variable *a*. The conditional probability distribution of *a* for a given *x* is [22,23,24,25]

(4) $$P\left(a\;|\;x\right)\;=\;\frac{P(x,a)}{P(x)}$$

where P(x) is the marginal of *x*

(5) P(x)=∫P(x,a)da

The global average of *a* is

(6) $$\langle a\rangle =\;\int \int \;aP\left(x,a\right)dxda$$

and the conditional value of *a* for a fixed *x* is given by

(7) $${\langle a\rangle }_{x}=\int aP\left(a\;|\;x\right)da=\frac{1}{P(x)}\int aP\left(x,a\right)da$$

The relation between local and global averages is

(8) $$\langle a\rangle =\int {\langle a\rangle }_{x}P\left(x\right)dx$$

Now consider the relation between the global standard deviation, $\sigma_{a}^{2},$

(9) $$\sigma_{a}^{2}=\langle a^{2}\rangle -{\langle a\rangle }^{2}$$

and the conditional standard deviation, $\sigma_{a|x}^{2}$, given by

(10) $$\sigma_{a|x}^{2}={\langle a^{2}\rangle }_{x}-{\langle a\rangle }_{x}^{2}$$

Unlike Equation (8), it is not the case that the global standard deviation is the average of the conditional standard deviation, that is,

(11) $$\sigma_{a}^{2}\neq \int P\left(x\right)\sigma_{a|x}^{2}\;dx$$

The relation is [26]

(12) $$\sigma_{a}^{2}=\int \sigma_{a|x}^{2}\;P\left(x\right)\;dx\;+\int {\left({\langle a\rangle }_{x}-\langle a\rangle \right)}^{2}\;P\left(x\right)\;dx$$

Equation (12) is proven in Appendix A.

We point out that *x* in Equation (12) does not have to be position but can be any other random variable, say *b*, and hence, we can write

(13) $$\sigma_{a}^{2}=\int \sigma_{a|b}^{2}\;P\left(b\right)\;db\;+\int {\left({\langle a\rangle }_{b}-\langle a\rangle \right)}^{2}\;P\left(b\right)\;db$$

## 3. Quantum Local Value at a Given Position

We now show that one can find a quantum analog to Equations (8) and (12); this will allow us to define local values and their standard deviation in quantum mechanics [26]. In quantum mechanics, observables are represented by operators. The global average, $\langle A\rangle ,$ and the global standard deviation, (ΔA), are defined, respectively, by

(14) $$\langle A\rangle =\langle \psi \left|A\right|\psi \rangle$$

and

(15) $${\left(\Delta A\right)}^{2}=\langle \psi \left|A^{2}\right|\psi \rangle -{\langle \psi \left|A\right|\psi \rangle }^{2}$$

In Appendix A, we show that if we break up $\frac{A\psi }{\psi }$ into its real and imaginary parts

(16) $$\frac{A\psi }{\psi }={\left(\frac{A\psi }{\psi }\right)}_{R}\;+\;i{\left(\frac{A\psi }{\psi }\right)}_{I}\;$$

then one may write the global quantum mechanical uncertainty, that is, the standard deviation, as [26]

(17) $${\left(\Delta A\right)}^{2}=\int {\left(\frac{A\psi }{\psi }\right)}_{I}^{2}{\;|\psi \left(x\right)|}^{2}\;dx\;+\;\int {\left[\;{\left(\frac{A\psi }{\psi }\right)}_{R}\;-\;\langle A\rangle \;\right]}^{2}{\left|\psi \left(x\right)\right|}^{2}\;dx\;$$

Comparing Equation (12) with Equation (17),

(18) $$\begin{array}{cc} {\langle A\rangle }_{x} & =\;{\left(\frac{A\psi }{\psi }\right)}_{R} \end{array}$$

(19) $$\begin{array}{cc} {\left(\Delta A\right)}_{x}^{2} & ={\left(\frac{A\psi }{\psi }\right)}_{I}^{2} \end{array}$$

We take these to be the local values in position. Consistent with the concept of conditional value, we have

(20) $$\int {\left(\frac{A\psi }{\psi }\right)}_{R}{\left|\psi \left(x\right)\right|}^{2}\;dx=\langle A\rangle \;$$

which corresponds to the classical idea, Equation (8).

### Quantum Local Values at a Given Momentum

If φ(p) is the momentum wave function

(21) $$\varphi \left(p\right)=\frac{1}{\sqrt{2\pi \hbar }}\int \psi \left(x\right)e^{-ixp/\hbar }dx$$

then it follows that the standard deviation, ΔA, is given by

(22) $${\left(\Delta A\right)}^{2}=\int {\left(\frac{A\varphi }{\varphi }\right)}_{I}^{2}{\;|\varphi \left(p\right)|}^{2}\;dp\;+\;\int {\left[\;{\left(\frac{A\varphi }{\varphi }\right)}_{R}\;-\;\langle A\rangle \;\right]}^{2}{\left|\varphi \left(p\right)\right|}^{2}\;dp\;$$

where now the operator A is written in the momentum representation. Comparing Equation (22) with Equation (13), we see that we may define the local value of A for a given momentum and its conditional standard deviation by

(23) $${\langle A\rangle }_{p}={\left(\frac{A\varphi }{\varphi }\right)}_{R}$$

and

(24) $${\left(\Delta A\right)}_{|p}^{2}={\left(\frac{A\varphi }{\varphi }\right)}_{I}^{2}\;$$

Similar to Equation (20), we have

(25) $$\langle A\rangle \;=\int {\left(\frac{A\varphi }{\varphi }\right)}_{R}{\left|\varphi \left(p\right)\right|}^{2}\;dp\;$$

## 4. Local Values for Two Arbitrary Observables

In the above, we considered the local value at a given position and the local value at a given momentum. We now consider the case of two arbitrary variables represented by the operators A and B. Consider the eigenvalue problem for B

(26) $$Bu_{\beta }\left(x\right)=\beta u_{\beta }\left(x\right)$$

where β and $u_{\beta }\left(x\right)$ are the eigenvalues and eigenfunctions, respectively. Any wave function, ψ(x), can be expanded as

(27) $$\psi \left(x\right)=\int \eta \left(\beta \right)u_{\beta }\left(x\right)d\beta$$

with

(28) $$\eta \left(\beta \right)=\int \psi \left(x\right)u_{\beta }^{*}\left(x\right)dx$$

where η(β) is the wave function in the beta representation. The expectation value of A is

(29) $$\langle A\rangle =\langle \eta \left|A\right|\eta \rangle =\int \eta^{*}\left(\beta \right)A\eta \left(\beta \right)d\beta$$

and the standard deviation is given by

(30) $${\left(\Delta A\right)}^{2}=\langle \eta \left|A^{2}\right|\eta \rangle -{\langle \eta \left|A\right|\eta \rangle }^{2}=\int \eta^{*}\left(\beta \right){\left(A-\;\langle A\rangle \right)}^{2}\eta \left(\beta \right)d\beta$$

We break up Aη/η into its real and imaginary parts

(31) $$\frac{A\eta }{\eta }={\left(\frac{A\eta }{\eta }\right)}_{R}\;+\;i{\left(\frac{A\eta }{\eta }\right)}_{I}\;$$

then the quantum mechanical standard deviation may be expressed as (Appendix A)

(32) $${\left(\Delta A\right)}^{2}=\int {\left(\frac{A\eta }{\eta }\right)}_{I}^{2}{\;|\eta \left(\beta \right)|}^{2}\;d\beta \;+\;\int {\left[\;{\left(\frac{A\eta }{\eta }\right)}_{R}\;-\;\langle A\rangle \;\right]}^{2}{\left|\eta \left(\beta \right)\right|}^{2}\;d\beta \;$$

Comparing with Equation (13), we have that the local value of A for a given [26]

(33) $$\begin{array}{cc} {\langle A\rangle }_{\beta } & =\;{\left(\frac{A\eta (\beta )}{\eta (\beta )}\right)}_{R} \end{array}$$

(34) $$\begin{array}{cc} {\left(\Delta A\right)}_{\beta }^{2} & ={\left(\frac{A\eta (\beta )}{\eta (\beta )}\right)}_{I}^{2} \end{array}$$

In general, we have that

(35) $$\langle A\rangle =\int \;{\left(\frac{A\eta (\beta )}{\eta (\beta )}\right)}_{R}{\left|\eta \left(\beta \right)\right|}^{2}\;d\beta$$

## 5. Examples

### 5.1. Local Momentum

For the operator A, we take the momentum operator, p,

(36) $$p=\frac{\hbar }{i}\frac{d}{dx}$$

We have for Equation (17) that

(37) $$\left(\frac{p\psi }{\psi }\right)=\frac{\hbar }{2i}\left(\frac{1}{\psi }\frac{d}{dx}\psi -\frac{1}{\psi^{*}}\frac{d}{dx}\psi^{*}\right)+\frac{\hbar }{2i}\left(\frac{1}{\psi }\frac{d}{dx}\psi +\frac{1}{\psi^{*}}\frac{d}{dx}\psi^{*}\right)$$

The first term is real, and the second is purely imaginary; therefore,

(38) $$\begin{array}{cc} {\left(\frac{p\psi }{\psi }\right)}_{R} & =\frac{\hbar }{2i}\left(\frac{1}{\psi }\frac{d}{dx}\psi -\frac{1}{\psi^{*}}\frac{d}{dx}\psi^{*}\right) \end{array}$$

(39) $$\begin{array}{cc} {\left(\frac{p\psi }{\psi }\right)}_{I} & =-\frac{\hbar }{2}\left(\frac{1}{\psi }\frac{d}{dx}\psi +\frac{1}{\psi^{*}}\frac{d}{dx}\psi^{*}\right) \end{array}$$

Hence, the local value of momentum is

(40) $${\langle \;p\;\rangle }_{x}={\left(\frac{p\psi }{\psi }\right)}_{R}=\frac{\hbar }{2i}\left(\frac{1}{\psi }\frac{d}{dx}\psi -\frac{1}{\psi^{*}}\frac{d}{dx}\psi^{*}\right)$$

The usual formula for the quantum mechanical current, j(x), is obtained by way of

(41) $$j\left(x\right)={\langle \;p\;\rangle }_{x}{\left|\psi \right|}^{2}/m$$

giving

(42) $$j\left(x\right)=\frac{\hbar }{2mi}\left(\psi^{*}\left(x\right)\frac{d}{dx}\psi (x)-\psi \left(x\right)\frac{d}{dx}\psi^{*}\left(x\right)\right)$$

The conditional standard deviation is given by

(43) $${\left(\Delta p\right)}_{x}^{2}={\left(\frac{p\psi }{\psi }\right)}_{I}^{2}=\frac{\hbar^{2}}{4}{\left(\frac{1}{\psi }\frac{d}{dx}\psi +\frac{1}{\psi^{*}}\frac{d}{dx}\psi^{*}\right)}^{2}$$

If we express the wave function in terms of amplitude and phase,

(44) $$\;\psi \left(x\right)=R\left(x\right)e^{iS(x)/\hbar }$$

we have that

(45) $$\frac{p\psi }{\psi }=\frac{\hbar }{i}\frac{R^{\prime }\left(x\right)}{R(x)}+S^{\prime }\left(x\right)\;$$

and hence,

(46) $$\begin{array}{cc} {\left(\frac{p\psi }{\psi }\right)}_{R} & =S^{\prime }\left(x\right)\; \end{array}$$

(47) $$\begin{array}{cc} {\left(\frac{p\psi }{\psi }\right)}_{I} & =-\hbar \frac{R^{\prime }\left(x\right)}{R(x)} \end{array}$$

The conditional expectation value of momentum is given by

(48) $${\langle \;p\;\rangle }_{x}=S^{\prime }\left(x\right)$$

and the conditional standard deviation by

(49) $${\left(\Delta p\right)}_{x}^{2}=\hbar^{2}\;{\left(\frac{R^{\prime }\left(x\right)}{R(x)}\right)}^{2}$$

For the uncertainty of momentum, Δp, we have, using Equation (30):

(50) $${\left(\Delta p\right)}^{2}=\int \;{\left(\frac{p\psi }{\psi }\right)}_{I}^{2}\;R^{2}\left(x\right)dx\;+\int {\left(\;{\left(\frac{p\psi }{\psi }\right)}_{R}-\langle \;p\;\rangle \right)}^{2}R^{2}\left(x\right)\;dx\;$$

Explicitly,

(51) $${\left(\Delta p\right)}^{2}=\hbar^{2}\int \;{\left(\frac{R^{\prime }\left(x\right)}{R(x)}\right)}^{2}\;R^{2}\left(x\right)dx\;+\int {\left(\;S^{\prime }\left(x\right)-\langle \;p\;\rangle \right)}^{2}R^{2}\left(x\right)\;dx\;$$

We note that current is defined at each point in space and of course, there is an intimate connection between current and average momentum [1,2]. In particular, if the momentum wave function, φ(p), is

(52) $$\varphi \left(p\right)=\frac{1}{\sqrt{2\pi \hbar }}\int \psi \left(x\right)\;e^{-ixp/\hbar }dx$$

then the average momentum is given by

(53) $$\langle p\rangle =\int p\;{\left|\varphi \left(p\right)\right|}^{2}dp$$

and also by

(54) $$\langle p\rangle =\int S^{\prime }\left(x\right)\;{\left|\psi \left(x\right)\right|}^{2}dx$$

This shows that the average momentum can be obtained by its local value and the probability of obtaining it at that point,${\;|\psi \left(x\right)|}^{2}$. This is a special case of Equation (35). These results are similar to the time-frequency case, where one relates instantaneous frequency to the energy density spectrum [13,27,28,29,30].

### 5.2. Uncertainty Principle

The local momentum given by Equation (48),

(55) $${\langle p\rangle }_{x}=S^{\prime }\left(x\right)$$

does not, in general, commute with the momentum operator. The commutator of ${\langle \;p\;\rangle }_{x}$ and p is

(56) $$\left[S^{\prime }\left(x\right),p\right]=i\hbar S^{\prime \prime }\left(x\right)$$

Using the general uncertainty principle between any two operators A and B [1,2]

(57) $${\left(\Delta A\right)}^{2}{\left(\Delta B\right)}^{2}⩾\frac{1}{2}{\left|\langle \;\left[A,B\right]\;\rangle \right|}^{2}$$

we have that

(58) $${\left(\Delta \;p\right)}_{x}^{2}{\left(\Delta p\right)}^{2}⩾\frac{\hbar }{2}{\left|\langle S^{\prime \prime }\left(x\right)\rangle \right|}^{2}$$

## 6. Local Acceleration and Force

Following Landau and Lipchitz [31], we define the velocity and acceleration operator by

(59) $$v=\frac{p}{m}$$

and

(60) $$a=\frac{d}{dt}v=\frac{1}{m}\frac{d}{dt}p$$

For Hamiltonians of the form

(61) $$H=\frac{p^{2}}{2m}+V\left(x\right)$$

with real potential function, the Heisenberg equation of motion gives the well-known result that

(62) $$i\hbar \frac{d}{dt}p=\left[p,H\right]=-i\hbar \frac{\partial }{\partial x}V$$

Hence,

(63) $$\frac{a\psi }{\psi }=-\frac{1}{m}\frac{\partial }{\partial x}V\;$$

giving

(64) $$\begin{array}{cc} {\left(\frac{a\;\psi }{\psi }\right)}_{R} & =-\frac{1}{m}\frac{\partial }{\partial x}V\; \end{array}$$

(65) $$\begin{array}{cc} {\left(\frac{a\;\psi }{\psi }\right)}_{I} & =0 \end{array}$$

The conditional expectation value for acceleration at a given position is therefore

(66) $${\langle a\rangle }_{x}=-\frac{1}{m}\frac{\partial }{\partial x}V$$

and the conditional force is

(67) $${\langle \;F\;\rangle }_{x}=-\frac{\partial }{\partial x}V$$

where we have taken

(68) $${\langle \;F\;\rangle }_{x}=m{\langle a\rangle }_{x}$$

Also, we have that

(69) $$\;\frac{d}{dt}{\langle \;p\;\rangle }_{x}=-\frac{\partial }{\partial x}V$$

The conditional standard deviation as per Equation (65) is zero

(70) $${\left(\Delta a\right)}_{x}=0$$

for real potentials. For the global standard deviation of acceleration, we have that

(71) $$\begin{array}{cc} {\;(\Delta a)}^{2} & =\int {\left(\frac{a\psi }{\psi }\right)}_{I}^{2}{\;|\psi \left(x\right)|}^{2}\;dx\;+\;\int {\left[\;{\left(\frac{a\psi }{\psi }\right)}_{R}\;-\;\langle a\rangle \;\right]}^{2}{\left|\psi \left(x\right)\right|}^{2}\;dx\; \end{array}$$

(72) $$\begin{array}{cc} \; & =\;\int {\left[\;{\left(\frac{a\psi }{\psi }\right)}_{R}\;-\;\langle a\rangle \;\right]}^{2}{\left|\psi \left(x\right)\right|}^{2}\;dx\; \end{array}$$

which simplifies to

(73) $${\left(\Delta a\right)}^{2}={\left(\Delta \left(\frac{1}{m}\frac{\partial }{\partial x}V\right)\right)}^{2}$$

where

(74) $${\left(\Delta \left(\frac{1}{m}\frac{\partial }{\partial x}V\right)\right)}^{2}=\int {\left[\frac{1}{m}\frac{\partial }{\partial x}V\;-\;\langle \;\frac{1}{m}\frac{\partial }{\partial x}V\;\;\rangle \;\right]}^{2}{\left|\psi \left(x\right)\right|}^{2}\;dx\;$$

as expected.

## 7. Local Kinetic Energy

We use the above formulation to define local kinetic energy. We further discuss local kinetic energy and its historical development in Section 10.5.

For convenience, we define the kinetic energy operator, K, by

(75) $$K=\frac{p^{2}}{2m}$$

Straightforward calculation gives

(76) $$\frac{K\psi }{\psi }=-\frac{\hbar^{2}}{2m}\frac{R^{\prime \prime }}{R}+\frac{S^{\prime^{2}}}{2m}-i\frac{\hbar }{2m}\left[\frac{2R^{\prime }S^{\prime }}{R}+S^{\prime \prime }\right]$$

and therefore,

(77) $${\left(\frac{K\psi }{\psi }\right)}_{R}=-\frac{\hbar^{2}}{2m}\frac{R^{\prime \prime }}{R}+\frac{S^{\prime^{2}}}{2m}$$

(78) $${\left(\frac{K\psi }{\psi }\right)}_{I}=-\frac{\hbar }{2m}\left(\frac{2R^{\prime }\;S^{\prime }}{R}+S^{\prime \prime }\right)$$

Therefore, using Equation (18), the conditional average is

(79) $${\langle \;\;K\;\;\rangle }_{x}=-\frac{\hbar^{2}}{2m}\frac{R^{\prime \prime }}{R}+\frac{S^{\prime^{2}}}{2m}$$

and the conditional standard deviation is given by

(80) $${\left(\Delta K\right)}_{x}^{2}=\frac{\hbar^{2}}{4m^{2}}\;{\left(\frac{2R^{\prime }S^{\prime }}{R}+S^{\prime \prime }\right)}^{2}$$

For the global standard deviation, using Equation (17), we have that

(81) $${\left(\Delta K\right)}^{2}=\int {\left(\frac{\hbar }{2m}\right)}^{2}\int {\left(S^{\prime \prime }+2\frac{R^{\prime }S^{\prime }}{R}\right)}^{2}R^{2}\left(x\right)\;dx+\int {\left(\;\frac{1}{2m}\;S^{\prime^{2}}-\frac{\hbar^{2}}{2m}\frac{R^{\prime \prime }}{R}\;-\;\langle \;K\;\rangle \;\right)}^{2}R^{2}\left(x\right)\;dx$$

In rectangular coordinates,

(82) $$\begin{array}{cc} {\left(\frac{1}{\psi }K\psi \right)}_{R} & =-\frac{\hbar^{2}}{2m}\left(\frac{1}{\psi }\frac{d^{2}}{dx^{2}}\psi +\frac{1}{\psi^{*}}\frac{d^{2}}{dx^{2}}\psi^{*}\right) \end{array}$$

(83) $$\begin{array}{cc} {\left(\frac{1}{\psi }K\psi \right)}_{I} & =-\frac{\hbar^{2}}{2m}\left(\frac{1}{\psi }\frac{d^{2}}{dx^{2}}\psi -\frac{1}{\psi^{*}}\frac{d^{2}}{dx^{2}}\psi^{*}\right) \end{array}$$

Therefore,

(84) $${\langle \;\;K\;\;\rangle }_{x}=-\frac{\hbar^{2}}{2m}\left(\frac{1}{\psi }\frac{d^{2}}{dx^{2}}\psi +cc\right)$$

and

(85) $${\left(\Delta K\right)}_{|x}^{2}=\frac{\hbar^{2}}{4m^{2}}\;{\left[\frac{1}{\psi }\frac{d^{2}}{dx^{2}}\psi -cc\right]}^{2}$$

## 8. Local Energy and the Quantum Potential 

For the Hamiltonian

(86) $$H=\frac{p^{2}}{2m}+V\left(x\right)$$

we have that [26]

(87) $$\frac{H\psi }{\psi }=V\left(x\right)+\frac{1}{2m}\;S^{\prime^{2}}-\frac{\hbar^{2}}{2m}\frac{R^{\prime \prime }}{R}-i\frac{\hbar }{2m}\left(\;S^{\prime \prime }+2\frac{R^{\prime }S^{\prime }}{R}\right)$$

Therefore,

(88) $$\begin{array}{cc} {\left(\frac{H\psi }{\psi }\right)}_{R} & =V\left(x\right)+\frac{1}{2m}\;S^{\prime^{2}}-\frac{\hbar^{2}}{2m}\frac{R^{\prime \prime }}{R} \end{array}$$

(89) $$\begin{array}{cc} {\left(\frac{H\psi }{\psi }\right)}_{I} & =-\frac{\hbar }{2m}\left(\;S^{\prime \prime }+2\frac{R^{\prime }S^{\prime }}{R}\right)\; \end{array}$$

Hence, for the conditional value of energy for a given position, we have

(90) $${\langle \;H\;\rangle }_{x}=V\left(x\right)+\frac{1}{2m}\;S^{\prime^{2}}-\frac{\hbar^{2}}{2m}\frac{R^{\prime \prime }}{R}\;$$

and for the local standard deviations, we have

(91) $${\left(\Delta H\right)}_{|x}^{2}={\left(\frac{\hbar }{2m}\right)}^{2}{\left(\;S^{\prime \prime }+2\frac{R^{\prime }S^{\prime }}{R}\right)}^{2}\;$$

The last term in Equation (90) is the quantum potential as derived by Bohm [32,33,34,35]:

(92) $$Q=-\frac{\hbar^{2}}{2m}\frac{R^{\prime \prime }}{R}$$

The global standard deviation for the Hamiltonian is, therefore, given by

(93) $$\begin{array}{cc} {\left(\Delta H\right)}^{2} & =\int {\left(\frac{\hbar }{2m}\right)}^{2}\int {\left(S^{\prime \prime }+2\frac{R^{\prime }S^{\prime }}{R}\right)}^{2}R^{2}\left(x\right)\;dx\\ \; & +\int {\left(\;\frac{1}{2m}\;S^{\prime^{2}}+\;V\left(x\right)-\frac{\hbar^{2}}{2m}\frac{R^{\prime \prime }}{R}\;-\;\langle \;H\;\rangle \;\right)}^{2}R^{2}\left(x\right)\;dx \end{array}$$

### 8.1. Real Wave Functions

For real wave functions, S(x)=0, and hence,

(94) $${\langle H\rangle }_{x}=V\left(x\right)-\frac{\hbar^{2}}{2m}\frac{R^{\prime \prime }}{R}$$

and

(95) $${\left(\Delta H\right)}_{x}^{2}=0$$

The global standard deviation as per Equation (93) is then

(96) $${\left(\Delta H\right)}^{2}=\int {\left(\;V\left(x\right)-\frac{\hbar }{2m}\frac{R^{\prime \prime }}{R}\;-\;\langle \;H\;\rangle \;\right)}^{2}R^{2}\left(x\right)\;dx$$

### 8.2. Time-Dependent Wave Function

Writing Schrödinger’s equation as

(97) $$i\hbar \frac{\partial \psi }{\partial t}=H\psi$$

and using

(98) $$\;\psi \left(x\right)=R\left(x\right)e^{iS(x)/\hbar }$$

we developed in the above section the conditional average and standard deviation of H by considering the right-hand side of Equation (97). Now, we find expressions for the same quantities using the left-hand side Equation (97). Differentiating Equation (97) with respect to time, we consider in the above the right-hand side of the Schrödinger equation. We now consider the left-hand side.

Using Equation (98), we have that [26]

(99) $$i\hbar \frac{1}{\psi }\frac{\partial \psi }{\partial t}=\left[-\frac{\partial S}{\partial t}+i\hbar \frac{1}{R}\frac{\partial R}{\partial t}\right]$$

and hence,

(100) $$\begin{array}{cc} {\langle \;H\;\rangle }_{x} & ={\left(i\hbar \frac{1}{\psi }\frac{\partial \psi }{\partial t}\right)}_{R}=-\frac{\partial S}{\partial t} \end{array}$$

(101) $$\begin{array}{cc} {\left(\Delta H\right)}_{x}^{2} & =\hbar^{2}{\left(\frac{1}{R}\frac{\partial R}{\partial t}\;\right)}^{2} \end{array}$$

The standard deviation may be written as

(102) $${\left(\Delta H\right)}^{2}=\hbar^{2}\int \;{\left(\frac{1}{R}\frac{\partial R}{\partial t}\right)}^{2}\;R^{2}\left(x\right)\;dx\;+\int {\left(\frac{\partial S}{\partial t}\;+\langle \;H\;\rangle \right)}^{2}R^{2}\left(x\right)\;dx\;$$

### 8.3. Interpretation and Contrast with Bohm Theory

We now contrast our approach with that of standard Bohm theory [32,33,34,35,36]. First, we point out that if we equate the two expressions Equations (90) and (100) and also Equations (91) and(101), we obtain

(103) $$\begin{array}{cc} -\frac{\partial S}{\partial t} & =V\left(x\right)+\frac{1}{2m}\;S^{\prime^{2}}-\frac{\hbar^{2}}{2m}\frac{R^{\prime \prime }}{R} \end{array}$$

(104) $$\begin{array}{cc} \frac{1}{R}\frac{\partial R}{\partial t} & =-\frac{1}{2m}\left(\;S^{\prime \prime }+2\frac{R^{\prime }S^{\prime }}{R}\right) \end{array}$$

These are the basic equations of Bohm theory. In addition, from these equations, one can derive

(105) $$\frac{\partial R^{2}}{\partial t}=-\frac{1}{m}\frac{\partial }{\partial x}\left[R^{2}\frac{\partial S}{\partial x}\right]=-\frac{1}{m}\left[2R\;\frac{\partial S}{\partial x}\;\frac{\partial R}{\partial x}+R^{2}\;\frac{\partial^{2}S}{\partial x^{2}}\right]$$

In Bohm theory, one writes

(106) $$\;\;p\left(x,t\right)\;=S^{\prime }\left(x,t\right)$$

where p(x,t) is the momentum of a *particle*. In contrast, we write

(107) $${\langle \;p\;\rangle }_{x}=S^{\prime }\left(x,t\right)$$

which is a probabilistic statement and hence keeps the probabilistic aspect of quantum mechanics. Further, in Bohm theory, one writes Newton’s equation of motion for the particle as

(108) $$m\frac{d^{2}x}{dt^{2}}=-\frac{\partial }{\partial x}\left(Q+V\right)$$

where *Q* is the quantum potential.

(109) $$Q=-\frac{\hbar^{2}}{2m}\frac{1}{R}\left(\frac{\partial^{2}R}{\partial x^{2}}\right)$$

In our case, it arises in the expression for the conditional value of the Hamiltonian

(110) $${\langle \;H\;\rangle }_{x}={\left(\frac{H\psi }{\psi }\right)}_{R}=V\left(x\right)+\frac{1}{2m}\;S^{\prime^{2}}+Q\left(x\right)$$

which may be written as

(111) $${\langle \;H\;\rangle }_{x}={\left(\frac{H\psi }{\psi }\right)}_{R}=V\left(x\right)+\frac{{\langle \;p\;\rangle }_{x}^{2}}{2m}\;+Q\left(x\right)$$

The relation between Bohm theory and quasi-distributions has been investigated [37,38].

### 8.4. Local Equation of Motion

Starting with Heisenberg’s equation of motion

(112) $$i\hbar \frac{d}{dt}A=\left[A,H\right]$$

and using

(113) $$\begin{array}{cc} {\langle \left[A,H\right]\rangle }_{x} & =\;{\left(\frac{[A,H]\psi }{\psi }\right)}_{R} \end{array}$$

(114) $$\begin{array}{cc} {\left(\Delta \left[A,H\right]\right)}_{x}^{2} & ={\left(\frac{[A,H]\psi }{\psi }\right)}_{I}^{2} \end{array}$$

we have that

(115) $$i\hbar \frac{d}{dt}{\langle A\rangle }_{x}=\;{\left(\frac{[A,H]\psi }{\psi }\right)}_{R}$$

or

(116) $$i\hbar \frac{d}{dt}{\langle A\rangle }_{x}={\langle \left[A,H\right]\rangle }_{x}\;$$

Also, we have that the standard deviation of the commutator [A,H] is given by

(117) $${\left(\Delta \left[A,H\right]\right)}^{2}=\int {\left(\frac{[A,H]\psi }{\psi }\right)}_{I}^{2}{\;|\psi \left(x\right)|}^{2}\;dx\;+\;\int {\left[\;{\left(\frac{[A,H]\psi }{\psi }\right)}_{R}\;-\;\langle \;\;\left[A,H\right]\;\;\rangle \;\right]}^{2}{\left|\psi \left(x\right)\right|}^{2}\;dx\;$$

### 8.5. Local Ehrenfest Theorem

Equation (116) may be thought of as a generalization of the Ehrenfest theorem for arbitrary operators.

The standard Ehrenfest theorem for momentum is

(118) $$\;\frac{d}{dt}\langle \;p\;\rangle =-\frac{\partial }{\partial x}\langle \;V\left(x\right)\;\rangle$$

and involves global quantities 〈p〉 and 〈V(x)〉. In contrast, we have that

(119) $$\;\frac{d}{dt}{\langle \;p\;\rangle }_{x}=-\frac{\partial }{\partial x}V$$

To obtain the standard result, we integrate both sides of Equation (119) with ${\left|\psi \left(x\right)\right|}^{2}$ to obtain Equation (118).

### 8.6. Local Correlation

Bohm [1] introduced the idea of the correlation operator

(120) $$C=\frac{1}{2}\left(\mathrm{px}+\mathrm{xp}\right)$$

This operator is also related to the deletion or scale operator [39,40]. Direct calculation on the wave function gives [26]

(121) $$\frac{C\psi }{\psi }=xS^{\prime }\left(x\right)+\frac{\hbar }{i}\left(x\frac{R^{\prime }\left(x\right)}{R(x)}+\frac{1}{2}\right)$$

Therefore,

(122) $$\begin{array}{cc} {\left(\frac{C\psi }{\psi }\right)}_{R} & =xS^{\prime }\left(x\right) \end{array}$$

(123) $$\begin{array}{cc} {\left(\frac{C\psi }{\psi }\right)}_{I} & =-\hbar \left(x\frac{R^{\prime }\left(x\right)}{R(x)}+\frac{1}{2}\right) \end{array}$$

Hence, the local value of C is

(124) $${\langle C\rangle }_{x}=xS^{\prime }\left(x\right)$$

For the global uncertainty, we have

(125) $${\left(\Delta C\right)}^{2}=\hbar^{2}\int \;{\left(x\frac{R^{\prime }\left(x\right)}{R(x)}+\frac{1}{2}\right)}^{2}\;R^{2}\left(x\right)\;dx\;+\int {\left(\;x\;S^{\prime }\left(x\right)-{\langle C\rangle }_{x}\right)}^{2}R^{2}\left(x\right)\;dx$$

## 9. Quantum Group Delay: Position for a Given Momentum

We now consider the average position for a given momentum. In the case of signal processing, where we are dealing with time-dependent signals, this is called the group delay [13,41,42]. The position operator in the momentum representation is

(126) $$x=i\hbar \frac{d}{dp}$$

Denoting the momentum wave function by φ(p), we have that

(127) $${\left(\frac{x\varphi }{\varphi }\right)}_{R}=-\frac{1}{{\left|\varphi \right|}^{2}}\frac{\hbar }{2i}\left(\varphi^{*}\left(p\right)\frac{d}{dp}\varphi (p)-\varphi \left(p\right)\frac{d}{dp}\varphi^{*}\left(p\right)\right)$$

Alternatively, if we express the momentum wave function in terms of its phase and amplitude

(128) $$\varphi \left(p\right)=T\left(p\right)e^{iU(p)/\hbar }\;$$

then

(129) $$\frac{x\varphi }{\varphi }=-\frac{\hbar }{i}\frac{T^{\prime }\left(p\right)}{T(p)}-U^{\prime }\left(p\right)$$

Hence,

(130) $${\langle \;x\;\rangle }_{p}=-U^{\prime }\left(p\right)$$

We call ${\langle \;\;x\;\;\rangle }_{p}$ the quantum group delay (in analogy to the time-frequency case). The local standard deviation is

(131) $${\left(\Delta x\right)}_{p}^{2}=\hbar^{2}\;{\left(\frac{T^{\prime }\left(p\right)}{T(p)}\right)}^{2}$$

**Free particle**. Consider the general solution to the Schrödinger wave equation for a free particle

(132) $$\varphi \left(p,t\right)=\varphi \left(p,0\right)\exp\left[\frac{p^{2}}{2mi\hbar }t\right]$$

We have that

(133) $$\frac{d}{dp}\varphi \left(p,t\right)=\left[\frac{pt}{mi\hbar }\varphi \left(p,0\right)+\left(\frac{d}{dp}\varphi \left(p,0\right)\right)\right]\exp\left[\frac{p^{2}}{2mi\hbar }t\right]$$

giving that

(134) $$\frac{x\varphi (p,t)}{\varphi (p,t)}=\frac{p}{m}t+\frac{x\varphi (p,0)}{\varphi (p,0)}$$

Hence,

(135) $$\begin{array}{cc} {\left(\frac{x\varphi (p,t)}{\varphi (p,t)}\right)}_{R} & =\frac{p}{m}t+{\left(\frac{x\varphi (p,0)}{\varphi (p,0)}\right)}_{R} \end{array}$$

(136) $$\begin{array}{cc} {\left(\frac{x\varphi (p,t)}{\varphi (p,t)}\right)}_{I} & ={\left(\frac{x\varphi (p,0)}{\varphi (p,0)}\right)}_{I} \end{array}$$

This shows that the expected value of position for a given momentum at time *t* changes as

(137) $${\langle \;\;x\;\;\rangle }_{p}\left(t\right)=\frac{p}{m}t+{\langle \;\;x\;\;\rangle }_{p}\left(0\right)$$

Also, we have that the local standard deviation does not change:

(138) $${\left(\Delta \;x\right)}_{p}^{2}=0$$

As an example, if we take a Gaussian in momentum space to be

(139) $$\varphi \left(p,0\right)=\frac{1}{{\left(2\pi \sigma_{p}^{2}\right)}^{1/4}}e^{-\frac{{\left(p-b\right)}^{2}}{4\sigma_{p}^{2}}-ia{\left(p-b\right)}^{2}}$$

where a,b,$\sigma_{p}^{2}$ are arbitrary real parameters, we obtain

(140) $$\begin{array}{cc} {\left(\frac{x\varphi }{\varphi }\right)}_{R} & =\frac{p}{m}t+2\hbar a\left(p-b\right) \end{array}$$

(141) $$\begin{array}{cc} {\left(\frac{x\varphi }{\varphi }\right)}_{I} & =-\hbar \frac{\left(p-b\right)}{2\sigma_{p}^{2}} \end{array}$$

### Example: Energy for a Given Momentum

The Hamiltonian in momentum space is

(142) $$H=\frac{p^{2}}{2m}+V\left(i\hbar \frac{d}{dp}\right)$$

Operating on a momentum wave function, we have that

(143) $$\frac{H\varphi }{\varphi }=\frac{p^{2}}{2m}+\frac{1}{\varphi }V\left(i\hbar \frac{d}{dp}\right)\varphi$$

Writing the potential as

(144) $$V\left(x\right)=\frac{1}{2\pi }\int V_{k}\left(k\right)e^{ixk}dk$$

with

(145) $$V_{k}\left(k\right)=\int V\left(x\right)e^{-ixk}dx$$

then

(146) $$\frac{1}{\varphi }V\left(i\hbar \frac{d}{dp}\right)\varphi =\frac{1}{2\pi }\frac{1}{\varphi }\int V_{k}\left(k\right)\varphi \left(p-hk\right)dk$$

## 10. Other Approaches to Local Values

### 10.1. Quasi-Probability Approach

If we had a proper joint distribution of position and momentum, conditional values would be well defined. The Wigner distribution [13,43,44,45,46,47,48]

(147) $$W\left(x,p\right)=\frac{1}{2\pi }\int \psi^{*}\left(x-\frac{\hbar }{2}\tau \right)\;\;\psi \left(x+\frac{\hbar }{2}\tau \right)\;\;e^{-i\tau p}d\tau \;$$

acts in some respects as a position–momentum distribution, but is not manifestly positive, and hence, it is often called quasi-distribution. Historically, many other quasi-distributions have been proposed in quantum mechanics and signal processing. Among these are the Margenou–Hill, Rihaceck, Choi–Wiliams, and ZAM [15,16,49,50,51,52], among others.

All bilinear phase space distributions, C(x,p), may be characterized and generated by [53,54]

(148) $$C\left(x,p\right)=\frac{1}{4\pi^{2}}\;\int \int \int \;\psi *\left(u-\;\frac{\hbar }{2}\tau \right)\psi \left(u+\;\frac{\hbar }{2}\tau \right)\Phi \left(\theta ,\tau \right)\;e^{-i\theta x-i\tau p+i\theta u}\;d\theta \;d\tau \;du$$

where Φ(θ,τ) is the kernel function that characterizes the distribution. If one considers distribution for kernels that satisfy

(149) Φ(θ,0)=1Φ(0,τ)=1

the quantum mechanical marginals are obtained:

(150) $$\begin{array}{cc} \int C(x,p)\;dp\; & ={\left|\psi (x)\right|}^{2}\; \end{array}$$

(151) $$\begin{array}{cc} \int C(x,p)\;dx\; & ={\left|\varphi (p)\right|}^{2} \end{array}$$

where ${\left|\psi (x)\right|}^{2}\;$ is the probability distribution of position and ${\left|\varphi (p)\right|}^{2}$ is the probability distribution of momentum. There is an infinite number of distributions that satisfy these conditions; the Wigner distribution is obtained by choosing Φ(θ,τ)=1. Of fundamental importance is the calculation of quantum averages. If we have a classical function $g\left(x,p\right)$ and a corresponding operator $G\left(x,p\right)$, we want

(152) $$\int \psi^{*}\left(x,t\right)G\left(x,p\right)\psi \left(x,t\right)\;dx=\;\int \int \;g\left(x,p\right)C\left(x,p,t\right)\;dp\;dx$$

where the left-hand side is the quantum way of calculating a global expectation value average and the right-hand side is the standard way of calculating averages. For Equation (152) to hold, there must be a relation between $g\left(x,p\right)$ and $G\left(x,p\right)$, and this will be discussed in Section 10.3.

The conditional distributions are

(153) $$C\left(\;p\;|\;\;x\right)\;=\;\frac{C(\;x,\;p)}{{\left|\psi (x)\right|}^{2}\;}\;\;\;\;\;\;\;;\;\;\;\;\;\;\;\;C\left(\;x\;|\;\;p\right)\;=\;\frac{C(\;x,\;p)}{{\left|\varphi (p)\right|}^{2}}$$

**Local momentum.** For simplicity, we consider product kernels

(154) Φ(θ,τ)=Φ(θτ)

The local momentum is

(155) $${\langle \;\;p\;\;\rangle }_{x}=\int p\;C\left(\;p\;|\;\;x\right)\;dp$$

If we want this ${\langle \;p\;\rangle }_{x}$ to equal the derivative of the phase

(156) $${\langle \;p\rangle }_{\;x}\;=\;S^{\prime }\left(\;x\right)$$

we must choose distributions that satisfy

(157) $$\;\Phi \left(0\right)\;=\;1\;\;\mathrm{and}\;\Phi^{\prime }\left(0\right)\;=\;0$$

**Local standard deviation.** For the second moment ${\langle p^{2}\rangle }_{x}$, one obtains

(158) $${\langle p^{2}\rangle }_{x}\;=\;\frac{\hbar^{2}}{2}\left[\;1+4\;\Phi^{\prime \prime }\left(0\right)\;\right]{\left(\frac{R^{\prime }\left(\;x\right)\;}{R(\;x)\;}\right)}^{2}-\frac{\hbar^{2}}{2}\left[\;1-4\;\Phi^{\prime \prime }\left(0\right)\;\right]\frac{R^{\prime \prime }\left(\;x\right)\;}{R(\;x)\;}+S^{\prime^{2}}\left(\;x\right)\;$$

and for the local standard deviation, we have

(159) $$\sigma_{\;p|\;x}^{2}\;=\;\frac{\hbar^{2}}{2}\left[\;1+4\;\Phi^{\prime \prime }\left(0\right)\;\right]{\left(\frac{R^{\prime }\left(\;x\right)\;}{R(\;x)\;}\right)}^{2}-\frac{\hbar^{2}}{2}\left[\;1-4\;\Phi^{\prime \prime }\left(0\right)\;\right]\frac{R^{\prime \prime }\left(\;x\right)\;}{R(\;x)\;}$$

If we want ${\langle \;\;p^{2}\;\rangle }_{\;x}$ and $\sigma_{\;p|\;x}^{2}$ to be manifestly positive, then the choice

(160) $$\;\Phi^{\prime \prime }\left(0\right)\;=\frac{1}{4}\;$$

gives

(161) $$\begin{array}{cc} {\langle \;\;p^{2}\;\rangle }_{\;x}\; & =\hbar^{2}{\left(\frac{R^{\prime }\left(\;x\right)\;}{R(\;x)\;}\right)}^{2}+S^{\prime^{2}}\left(\;x\right) \end{array}$$

(162) $$\begin{array}{cc} \sigma_{\;p|\;x}^{2}\; & =\hbar^{2}{\left(\;\frac{R^{\prime }\left(\;x\right)\;}{R(\;x)\;}\;\right)}^{2} \end{array}$$

which are both manifestly positive. Equation (162) for $\sigma_{\;p|\;x}^{2}$ is identical to the result obtained previously, Equation (49). We emphasize that there are an infinite number of distributions that satisfy Equations (157) and (160).

**Wigner distribution**. Moyal was the first to consider local moments for the Wigner distribution. The Wigner distribution case is obtained by taking the kernel to be one, Φ(θτ)=1. One obtains from the above equations that for the Wigner distribution

(163) $${\langle \;\;p\;\rangle }_{\;x}\;=\;S^{\prime }\left(\;x\right)$$

and

(164) $$\sigma_{p|x}^{2}=\frac{\hbar^{2}}{2}\left[\;{\left(\frac{R^{\prime }\left(x\right)\;}{R(x)\;}\right)}^{2}-\frac{R^{\prime \prime }\left(x\right)\;}{R(x)\;}\;\right]$$

The local standard deviation, $\sigma_{p|x}^{2}\;$, is not generally manifestly positive and cannot, in general, be interpreted.

### 10.2. Window Wave Function Approach

The window function approach for obtaining quasi-distributions gives interesting results and may be thought of as a way of estimating quantities [13,55,56]. One focuses on the position $x=x^{\prime }$ by multiplying the wave function by a relatively narrow function centered at $x^{\prime }.$ In particular, for the wave function ψ(x), which is the window function $W(x^{\prime }-x)$, the joint position momentum probability is

(165) $$P\left(x,p\right)\;={\left|\frac{1}{\sqrt{2\pi }}\int e^{-ipx^{\prime }/h}\psi \left(x^{\prime }\right)W^{*}\left(x^{\prime }-x\right)dx^{\prime }\;\right|}^{2}$$

This distribution is manifestly positive but does not satisfy the quantum mechanical marginals. The position marginals are given by

(166) $$P\left(x\right)=\int P\left(x,p\right)\;dp\;=\;\int {\left|\psi (x^{\prime })\right|}^{2}{\left|W(x^{\prime }-x)\right|}^{2}dx^{\prime }\;$$

(167) $$P\left(p\right)=\int P\left(x,p\right)\;dx\;=\;\int |\varphi \left(p^{\prime }\right){|}^{2}{\left|w(p^{\prime }-p)\right|}^{2}\;dp^{\prime }$$

where w(p) is the momentum wave function of the window W(x)

(168) $$w\left(p\right)=\frac{1}{\sqrt{2\pi h}}\int W\left(x\right)e^{-ixp/h}dx$$

We write the wave function and window in terms of their respective amplitude and phases

(169) $$\psi \left(x\right)=R\left(x\right)e^{iS(x)/h}\;\;\;\;\;\;\;\;\;\;\;;\;\;\;\;\;\;W\left(x\right)=R_{W}\left(x\right)e^{iS_{W}\left(x\right)/h}$$

then the first conditional moment of momentum for a given position is

(170) $${\langle p\rangle }_{x}\;=\frac{1}{P(x)}\;\int p\;P\left(x,p\right)\;dp$$

calculated to be [13]

(171) $${\langle \;p\;\rangle }_{x}\;=\;\frac{1}{P(x)}\int R^{2}\left(x^{\prime }\right)\;R_{W}^{2}\left(x^{\prime }-x\right)\;\left(\;S^{\prime }\left(x^{\prime }\right)\;+\;S_{W}^{\prime }\left(x^{\prime }-x\right)\;\right)\;dx^{\prime }$$

To obtain increasing position localization, we narrow the window so that

(172) $$R_{W}^{2}\left(x\right)\;\to \;\delta \left(x\right)\;$$

Then,

(173) $${\langle \;p\;\rangle }_{x}\;\to \frac{\int R_{\psi }^{2}\left(x^{\prime }\right)\;\delta \left(x^{\prime }-x\right)\;S^{\prime }\left(x^{\prime }\right)dx^{\prime }}{\int R_{\psi }^{2}\left(x^{\prime }\right)\;\delta \left(x^{\prime }-x\right)dx^{\prime }}=\frac{R_{\psi }^{2}\left(x\right)\;S^{\prime }\left(x\right)}{R_{\psi }^{2}\left(x\right)}=\;S^{\prime }\left(x\right)$$

That is,

(174) $${\langle \;p\;\rangle }_{x}\;\to \;S^{\prime }\left(x\right)$$

For the standard deviation, we know it has to go to infinity because we have narrowed the window to a delta function

(175) $$\sigma_{p|x}\to \infty$$

However, it goes to infinity in an interesting way, namely

(176) $$\;\sigma_{p|x}^{2}∼\hbar^{2}{\left(\frac{R^{\prime }}{R}\right)}^{2}\;\;+\;\;\left[\;\;\mathrm{window}\;\mathrm{dependent}\;\mathrm{terms}\to \infty \;\right]$$

The first term is identical to the conditional spread in momentum for a given position obtained as derived previously, Equation (49).

### 10.3. Correspondence Rules Approach

As mentioned in Section 10.1 for space phase averaging of a classical function g(x,p) to give the quantum results to the same result as the quantum result,

(177) $$\int \psi^{*}\left(x,t\right)G\left(x,p\right)\psi \left(x,t\right)\;dx=\;\int \int \;g\left(x,p\right)C\left(x,p,t\right)\;dp\;dx$$

where $G\left(x,p\right)$ is the quantum operator corresponding to the classical $g\left(x,p\right)$, there must be a relation between the classical function and quantum operator. Such relations have historically been called correspondence rules, or rules of association [57,58,59,60,61,62].

All correspondence rules may be characterized by taking the operator to be

(178) $$G_{\Phi }\left(x,p\right)\;=\;\int \int \;\hat{g}\left(\theta ,\tau \right)\Phi \left(\theta ,\tau \right)\;e^{i\theta x+i\tau p}\;d\theta \;d\tau$$

where Φ(θ,τ) is a two-dimensional function, called the kernel, as described in Section 10.1. By choosing different kernels, different rules are obtained. In Equation (178), $\hat{g}\left(\theta ,\tau \right)$ is the Fourier transform of the classical function g(x,p),

(179) $$\hat{g}\left(\theta ,\tau \right)\;=\;\frac{1}{4\pi^{2}}\;\int \int \;g\left(x,p\right)\;e^{-i\theta x-i\tau p}\;dx\;dp$$

with

(180) $$g\left(x,p\right)=\;\int \int \;\hat{g}\left(\theta ,\tau \right)\;\;e^{i\theta x+i\tau p}\;d\theta \;d\tau$$

Using the Baker–Housdorf theorem, Equations (178) may be written in the following alternate forms [62,63]:

(181) $$\begin{array}{cc} G_{\Phi }\left(x,p\right)\; & =\;\int \int \;\hat{g}\left(\theta ,\tau \right)\Phi \left(\theta ,\tau \right)\;e^{i\theta \tau \hbar /2}\;e^{i\theta x}\;e^{i\tau p}\;d\theta \;d\tau \end{array}$$

(182) $$\begin{array}{cc} \; & \;=\;\int \int \;\hat{g}\left(\theta ,\tau \right)\Phi \left(\theta ,\tau \right)\;e^{-i\theta \tau \hbar /2}\;e^{i\tau p}\;e^{i\theta x}\;d\theta \;d\tau \end{array}$$

The operation on a position and momentum wave function is given by

(183) $$G_{\Phi }\left(x,p\right)\;\psi \left(x\right)=\;\int \int \;\hat{g}\left(\theta ,\tau \right)\Phi \left(\theta ,\tau \right)\;e^{i\theta \tau \hbar /2}\;e^{i\theta x}\;\;\psi \left(x+\tau \hbar \right)d\theta \;d\tau$$

and

(184) $$G_{\Phi }\left(x,p\right)\;\varphi \left(p\right)=\;\int \int \;\hat{g}\left(\theta ,\tau \right)\Phi \left(\theta ,\tau \right)\;e^{-i\theta \tau \hbar /2}\;e^{i\tau p}\;\;\;\varphi \left(p-\theta \hbar \right)d\theta \;d\tau$$

Therefore,

(185) $$\begin{array}{cc} \frac{G_{\Phi }\left(x,p\right)\;\psi \left(x\right)}{\psi (x)} & =\frac{1}{\psi (x)}\;\int \int \;\hat{g}\left(\theta ,\tau \right)\Phi \left(\theta ,\tau \right)\;e^{i\theta \tau \hbar /2}\;e^{i\theta x}\;\;\psi \left(x+\tau \right)d\theta \;d\tau \end{array}$$

(186) $$\begin{array}{cc} \frac{G_{\Phi }\left(x,p\right)\;\varphi \left(p\right)}{\varphi (p)} & =\frac{1}{\varphi (p)}\;\int \int \;\hat{g}\left(\theta ,\tau \right)\Phi \left(\theta ,\tau \right)\;e^{-i\theta \tau \hbar /2}\;e^{i\tau p}\;\;\;\varphi \left(p-\theta \hbar \right)d\theta \;d\tau \end{array}$$

Hence, we have that

(187) $${\langle \;\;G_{\Phi }\left(x,p\right)\;\;\rangle }_{x}=\;{\left(\frac{G_{\Phi }\left(x,p\right)\;\psi \left(x\right)}{\psi (x)}\right)}_{R}$$

Also, the conditional average for a given momentum is

(188) $${\langle \;\;G_{\Phi }\left(x,p\right)\;\;\rangle }_{p}=\;{\left(\frac{G_{\Phi }\left(x,p\right)\;\varphi \left(p\right)}{\varphi (p)}\right)}_{R}$$

### 10.4. Characteristic Function Approach

We now approach the question of local values from the characteristic function approach point of view. For a probability distribution in two variables, P(a,b) the characteristic function, M(θ,τ), is defined:

(189) $$M\left(\theta ,\tau \right)\;=\;\int \int \;e^{i\theta a+i\tau b}P\left(a,b\right)\;dadb$$

Inversely, we have

(190) $$P\left(a,b\right)\;\;=\;\frac{1}{4\pi^{2}}\;\int \int \;M\left(\theta ,\tau \right)e^{-i\theta a-i\tau b}\;d\theta \;d\tau$$

The characteristic function is an expectation value, namely the expectation value of $e^{i\theta a+i\tau b}$

(191) $$M\left(\theta ,\tau \right)=\langle e^{i\theta a+i\tau b}\rangle$$

Now consider the average conditional value of *a* at a given b,

(192) $${\langle a\rangle }_{b}=\frac{1}{P(b)}\int P\left(a,b\right)\;da$$

where P(b) is the marginal of b. Substituting Equation (190) into Equation (192), one obtains

(193) $${\langle a\rangle }_{b}=\frac{1}{4\pi^{2}}\frac{1}{P(b)}\;\int \int \int \;aM\left(\theta ,\tau \right)e^{-i\theta a-i\tau b}dad\theta d\;\tau$$

which evaluates to

(194) $${\langle a\rangle }_{b}=\frac{1}{2\pi i}\frac{1}{P(b)}\int {\left.\frac{\partial M(\theta ,\tau )}{\partial \theta }\right|}_{\theta =0}\;e^{-i\tau b}d\;\tau$$

We now attempt to write the quantum mechanical equivalent to Equation (194). Since M(θ,τ) is an expectation value, we write

(195) $$M\left(\theta ,\tau \right)=\int \psi^{*}\left(x\right)\;M(\theta ,\tau )\psi \left(x\right)dx=\langle M(\theta ,\tau )\rangle$$

where M(θ,τ) is the operator corresponding to the classical M(θ,τ). Therefore, for the quantum case, we have

(196) $${\langle A\rangle }_{b}=\frac{1}{2\pi i}\frac{1}{P(b)}\int \langle {\left.\frac{\partial M(\theta ,\tau )}{\partial \theta }\right|}_{\theta =0}\rangle e^{-i\tau b}\;d\tau \;$$

There are an infinite number of expressions for M(θ,τ) because of the non-commutativity of the operators. For example, $M(\theta ,\tau )↔e^{i\theta A+i\tau B}$ or $e^{i\theta A}\;e^{i\tau B}$ or $e^{i\theta A/2}e^{i\tau B}e^{i\theta A/2}$ among others.

If we take *b* to be position, *x*, and take

(197) $$M\left(\theta ,\tau \right)=\frac{1}{2}{\left[e^{i\theta A},e^{i\tau x}\right]}_{+}$$

then

(198) $${\left.\frac{\partial M(\theta ,\tau )}{\partial \theta }\right|}_{\theta =0}=\frac{i}{2}{\left[A,e^{i\tau x}\right]}_{+}$$

which gives

(199) $${\langle A\rangle }_{x}=\frac{1}{4\pi }\frac{1}{{\left|\psi (x)\right|}^{2}}\frac{i}{2}\;\int \int \;\psi^{*}\left(x^{\prime }\right){\left[A,e^{i\tau x}\right]}_{+}\psi \left(x^{\prime }\right)e^{-i\tau b}\;d\alpha dx^{\prime }$$

Evaluation leads to

(200) $${\langle A\rangle }_{x}={\left(\frac{A\psi }{\psi }\right)}_{R}$$

which is the same answer as Equation (18). This was obtained in [64] by a different approach. Note that there are an infinite number of distributions that give this result.

### 10.5. Local Kinetic Energy—Various Expressions

As discussed in the introduction, local kinetic energy has played an import role in a number of areas of quantum mechanics. Four expressions that have been used by various authors are [5,8,9,10,64,65]

(201) $$\begin{array}{cc} K_{A} & =-\frac{\hbar^{2}}{2m}\psi^{*}\nabla^{2}\psi \end{array}$$

(202) $$\begin{array}{cc} K_{B} & =\frac{\hbar^{2}}{2m}{\left|\nabla \psi \right|}^{2} \end{array}$$

(203) $$\begin{array}{cc} K_{C} & =-\frac{\hbar^{2}}{4m}\left[\psi^{*}\nabla^{2}\psi +\psi \nabla^{2}\psi^{*}\right] \end{array}$$

(204) $$\begin{array}{cc} K_{D} & =\frac{\hbar^{2}}{2m}\left[{\left|\nabla \psi \right|}^{2}-\frac{1}{4}\nabla^{2}\left|\psi^{2}\right|\right] \end{array}$$

In polar coordinates (in one dimension), these expressions are

(205) $$\begin{array}{cc} K_{A} & =\frac{\hbar^{2}}{2m}\left[{\left(\frac{R^{\prime }}{R}\right)}^{2}+S^{\prime^{2}}-\frac{1}{R^{2}}\frac{d}{dx}RR^{\prime }+i\left[\frac{2R^{\prime }S^{\prime }}{R}+S^{\prime \prime }\right]\right] \end{array}$$

(206) $$\begin{array}{cc} K_{B} & =\frac{\hbar^{2}}{2m}\left[S^{\prime^{2}}+{\left(\frac{R^{\prime }}{R}\right)}^{2}\right]R^{2} \end{array}$$

(207) $$\begin{array}{cc} K_{C} & =\frac{\hbar^{2}}{2m}\left[S^{\prime^{2}}+{\left(\frac{R^{\prime }}{R}\right)}^{2}-\frac{1}{R^{2}}\frac{d}{dx}RR^{\prime }\right]R^{2} \end{array}$$

(208) $$\begin{array}{cc} K_{D} & =\frac{\hbar^{2}}{2m}\left[S^{\prime^{2}}+{\left(\frac{R^{\prime }}{R}\right)}^{2}-\frac{1}{4R^{2}}\frac{d^{2}}{dx^{2}}R^{2}\right]R^{2} \end{array}$$

All these local expressions give the correct quantum average kinetic energy

(209) $$\begin{array}{cc} \int K_{A,B,C,D}dx & =-\frac{\hbar^{2}}{2m}\int \psi^{*}\frac{d^{2}}{dx^{2}}\psi dx \end{array}$$

(210) $$\begin{array}{cc} \; & \;=\frac{\hbar^{2}}{2m}\int {\left|\frac{d}{dx}\psi \right|}^{2}dx \end{array}$$

Now

(211) $$\frac{\hbar^{2}}{2m}{\left|\frac{d}{dx}\psi \right|}^{2}=\frac{\hbar^{2}}{2m}\left[S^{\prime^{2}}+{\left(\frac{R^{\prime }}{R}\right)}^{2}\right]R^{2}$$

and it is only these terms that contribute to the global kinetic energy; the other local terms integrate to zero.

### 10.6. Formant Bandwidth

In the field of speech analysis, the concept of formant bandwidth, that is, the conditional standard deviations, has been developed [66,67,68]. From the point of view of the methods we have developed for position wave functions, in contrast to the signal analysis case, which deals with time functions, the bandwidth is taken to be of the form

(212) $$\sigma_{p|x}∼ae^{-B(x-x_{0})}$$

where *a* and *B* are constants.

In our formulation, the bandwidth for momentum at a given position *x* is Equation (49), where in this section, we take ℏ=1.

(213) $$\sigma_{p|x}=\;\frac{R^{\prime }\left(x\right)}{R(x)}$$

To show under what conditions Equation (213) can be approximated by Equation (212), we expand *R* and *S* in the wave function

(214) $$\;\psi \left(x\right)=R\left(x\right)e^{iS(x)/\hbar }$$

by a Taylor series

(215) $$\begin{array}{cc} R(x) & ∼R\left(x_{0}\right)+R^{\prime }\left(x_{0}\right)\left(x-x_{0}\right)+\cdots \end{array}$$

(216) $$\begin{array}{cc} S(x) & ∼S\left(x_{0}\right)+S^{\prime }\left(x_{0}\right)\left(x-x_{0}\right)+\cdots \end{array}$$

Therefore,

(217) $$\begin{array}{cc} \;\psi (x) & ∼\left[R\left(x_{0}\right)+R^{\prime }\left(x_{0}\right)\left(x-x_{0}\right)\right]e^{i\left[S\left(x_{0}\right)+S^{\prime }\left(x_{0}\right)\left(x-x_{0}\right)\right]/\hbar } \end{array}$$

(218) $$\begin{array}{cc} \; & \;=R\left(x_{0}\right)\left[1+\;\frac{R^{\prime }\left(x_{0}\right)}{R(x_{0})}\left(x-x_{0}\right)\right]e^{iS\left(x_{0}\right)+S^{\prime }\left(x_{0}\right)\left(x-x_{0}\right)/\hbar } \end{array}$$

If we want to fit the amplitude by

(219) $$ae^{-B(x-x_{0})}∼a\left(1-B(x-x_{0})\right)$$

then comparing Equation (218) with Equation (219), we have that

(220) $$\begin{array}{cc} a & =R(x_{0}) \end{array}$$

(221) $$\begin{array}{cc} B & =-\frac{R^{\prime }\left(x_{0}\right)}{R(x_{0})} \end{array}$$

This will be the case if the amplitude is very slowly varying.

### 10.7. Weak Value Approach

The concept of weak values originated with Aharanov et al. [69,70,71] and has been discussed in the contexts of quantum mechanical measurement theory. The possible relation of weak values to conditional values has been discussed by a number of authors [64,72,73,74]. Hiley [75] has given an interesting derivation of weak values. A paper relating the Hiley approach to weak values and the conditional values we have discussed in previous sections is being written [76]. Here we briefly show how the Hiley method may be modified to obtain Equation (18). For the operator A, we write the eigenvalue problem

(222) $$Au_{\alpha }\left(x\right)=\alpha u_{\alpha }\left(x\right)$$

where α and $u_{\alpha }\left(x\right)$ are the eigenvalues and eigenfunctions, respectively. The wave function can be expanded as

(223) $$\psi \left(x\right)=\int \eta \left(\alpha \right)u_{\alpha }\left(x\right)d\alpha$$

with

(224) $$\eta \left(\alpha \right)=\int \psi \left(x\right)u_{\alpha }^{*}\left(x\right)dx$$

where η(α) is the wave function in the alpha representation. Start with the expectation value in the alpha representation

(225) $$\langle A\rangle =\int \eta^{*}\left(\alpha \right)A\eta \left(\alpha \right)d\alpha$$

Following Hiley, substitute just for $\eta^{*}\left(\alpha \right)$, and multiplying and dividing the integrand by ψ(x), we have

(226) $$\begin{array}{cc} \langle A\rangle & =\;\int \int \;\psi^{*}\left(x\right)u_{\alpha }\left(x\right)A\eta \left(\alpha \right)d\alpha dx \end{array}$$

(227) $$\begin{array}{cc} \; & \;=\int {\left|\psi (x)\right|}^{2}\frac{\int \left[u_{\alpha }\left(x\right)A\eta \left(\alpha \right)d\alpha \right]}{\psi (x)}dx \end{array}$$

The weak value, a(x), is

(228) $$a\left(x\right)=\frac{1}{\psi (x)}\int u_{\alpha }\left(x\right)A\eta \left(\alpha \right)d\alpha$$

Now this can be simplified further. Substituting for η(α) as per Equation (224)

(229) $$\begin{array}{cc} \;a(x) & =\frac{1}{\psi (x)}\;\int \int \;u_{\alpha }\left(x\right)A\psi \left(x^{\prime }\right)u_{\alpha }^{*}\left(x^{\prime }\right)dx^{\prime }d\alpha \end{array}$$

(230) $$\begin{array}{cc} \; & =\frac{1}{\psi (x)}\int \delta \left(x-x^{\prime }\right)A\psi \left(x^{\prime }\right)dx^{\prime } \end{array}$$

or

(231) $$a\left(x\right)=\frac{A\psi (x)}{\psi (x)}$$

In some sense one can simplify the derivation even further. We have

(232) $$\begin{array}{cc} \langle A\rangle & =\int \psi^{*}\left(x\right)A\psi \left(x\right)dx \end{array}$$

(233) $$\begin{array}{cc} \; & \;=\int {\left|\psi (x)\right|}^{2}\frac{A\psi (x)}{\psi (x)}dx \end{array}$$

which gives Equation (231). The weak value, a(x), is generally complex and we write as per Equation (16)

(234) $$a\left(x\right)={\left(\frac{A\psi }{\psi }\right)}_{R}\;+\;i{\left(\frac{A\psi }{\psi }\right)}_{I}\;.$$

The expected value of a(x) is given by

(235) $$\langle a(x)\rangle =\int {\left|\psi (x)\right|}^{2}\left\{{\left(\frac{A\psi }{\psi }\right)}_{R}\;+\;i{\left(\frac{A\psi }{\psi }\right)}_{I}\;\right\}dx$$

If A is Hermitian, then we know that $\langle a(x)\rangle$ is real, and hence,

(236) $$\begin{array}{cc} \langle a(x)\rangle & =\int {\left|\psi (x)\right|}^{2}{\left(\frac{A\psi }{\psi }\right)}_{R}\;\;dx \end{array}$$

(237) $$\begin{array}{cc} 0 & =\int {\left|\psi (x)\right|}^{2}{\left(\frac{A\psi }{\psi }\right)}_{I}\;dx \end{array}$$

The real part of a(x) is what we have previously derived, and which we called the conditional value of A at a given *x*

(238) $${\langle A\rangle }_{x}={\left(\frac{A\psi }{\psi }\right)}_{R}$$

which is Equation (18).

## 11. Conclusions

We discuss the fundamental problem with the concept of conditional values in quantum mechanics. For the classical case where local value is defined by

(239) $${\langle a\rangle }_{x}=\int aP\left(a\;|\;x\right)da=\frac{1}{P(x)}\int aP\left(x,a\right)da$$

and where P(x,a) is a proper distribution function; then of course, global averages are given by

(240) $$\langle a\rangle =\int {\langle a\rangle }_{x}P\left(x\right)dx$$

where P(x) is the probability of x, which is the marginal of P(x,a). However, it is important to appreciate that the converse is not true. That is, for an ${\langle a\rangle }_{x}$ that satisfies Equation (240), it is not necessarily true that ${\langle a\rangle }_{x}$ is proper. By proper, we mean that it comes from some legitimate joint distribution. In analogy with other fields, if ${\langle a\rangle }_{x}$ is proper, we call it “representable” (or realizable); otherwise, we call it not representable. Even in standard probability theory, one comes across non-representable quantities. It is sometimes easy to see that a conditional is not representable. For example, if we are given ${\langle \;\;a^{2}\;\;\rangle }_{x}$, which is not manifestly positive, then obviously, it is not representable.

It is a remarkable fact that in quantum mechanics, we almost always deal with improper local quantities but that are nonetheless very useful. For the conditional values defined in quantum mechanics, ${\langle A\rangle }_{x},$ one traditionally checks that it indeed integrates to the global quantum mechanical average,

(241) $$\int {\langle A\rangle }_{x}{\left|\psi \left(x\right)\right|}^{2}\;dx=\langle A\rangle \;$$

However, Equation (241) is far from sufficient to establish the representability of ${\langle A\rangle }_{x}.$ To illustrate the issues, we consider the example of quantum mechanical current with unit mass

(242) $$j\left(x\right)=\frac{\hbar }{2i}\left(\psi^{*}\left(x\right)\frac{d}{dx}\psi (x)-\psi \left(x\right)\frac{d}{dx}\psi^{*}\left(x\right)\right)$$

We have that

(243) $${\int j\left(x\right)|\psi \left(x\right)|}^{2}\;dx=\langle p\rangle$$

where 〈p〉 is the average momentum given by

(244) $$\langle p\rangle ={\int p|\varphi \left(p\right)|}^{2}dp$$

where ${\left|\varphi \left(p\right)\right|}^{2}$ is the probability distribution of momentum. Quantum mechanical current plays a basic role in quantum mechanics, and often fits our intuitive classical notions. But it has peculiar properties that clearly indicate that it is not representable. In particular, Bracken and Melloy [77] showed that one can have wave functions where current is flowing out of a particular region, while there is an increase in probability in that region. Other peculiar behaviors of current are with the time of arrival issues [78,79,80]. A particularly dramatic example [81] that is totally in conflict with our intuition is that for momentum distributions, ${\left|\varphi \left(p\right)\right|}^{2}$, that are limited in range between two values, the current may range outside these values! That could not happen if the current was representable. These curious behaviors of current are generally not discussed in textbooks. The classic book by Schiff [82] does mention that the concept of current has limited applicability, but it is not clear what the limitations are. These issues are identical to the concept of “instantaneous frequency” in the field of signal processing [13,27,28,83,84,85].

The same issues discussed above apply to the general case. In particular, for an arbitrary operator, the eigenvalue problem

(245) $$Au_{\alpha }\left(x\right)=\alpha u_{\alpha }\left(x\right)$$

gives the numerical values that can be measured, namely the eigenvalues, α. The wave function in the α representation is given by

(246) $$\eta \left(\alpha \right)=\int \psi \left(x\right)u_{\alpha }^{*}\left(x\right)dx$$

with

(247) $$\psi \left(x\right)=\int \eta \left(\alpha \right)u_{\alpha }\left(x\right)dx$$

It is one of the main results of quantum mechanics that the average is given. Now consider the expected value of alpha

(248) $$\langle \alpha \rangle =\int \alpha {\left|\eta \left(\alpha \right)\right|}^{2}d\alpha =\int \psi^{*}\left(x\right)A\psi \left(x\right)dx$$

We have shown that a reasonable expression for local value is ${\left(A\psi /\psi \right)}_{R}$, and it follows that

(249) $$\langle \alpha \rangle ={\int \alpha |\eta \left(\alpha \right)|}^{2}d\alpha =\int {\left(\frac{A\psi }{\psi }\right)}_{R}\;{\left|\psi \left(x\right)\right|}^{2}dx$$

This is in analogy to Equation (240) for current. We believe that this expression and others for conditional values show peculiarities because of their non-representability, but these peculiarities have not been studied in general, and it would be very interesting to do so.

Another important issue regarding local values is that while there are many different local value expressions for a given operator, only some are used with profit. For example, the various expressions given in Section 10.5 for local kinetic energy are all different, and in fact, there are an infinite number of them. However, different expressions have been used for different purposes with profit. For example, a particular class of expressions is used to define a virial theorem in a region, which, in turn, has been used to define atoms in molecules. Not all local kinetic energy expressions work. On the other hand, *different* expressions have been profitably used in developing classical types of kinetic equations.

From a mathematical point of view, it is clear why there are an infinite number of local values for a given operator. What is not clear is why some are useful, and others are not. That is a subject worth exploring.

## Data Availability

The original contributions presented in the study are included in the article, further inquiries can be directed to author.

## Appendix A. Relation between Global and Conditional Standard Deviation

We derive the relationship between the global and conditional quantities for both the classical and quantum cases. We streamline the derivation given in [26]. Although we have derived parts of the derivation in the above, we make this appendix self-contained. We deal with the classical case first. For a probability distribution, P(x,a), of an arbitrary variable, a, and position *x*, the global average of *a* is given by

(A1) $$\langle a\rangle =\;\int \int \;aP\left(x,a\right)dxda$$

and the conditional average by

(A2) $${\langle a\rangle }_{x}=\int aP\left(a\;|\;x\right)da=\frac{1}{P(x)}\int aP\left(x,a\right)da$$

where P(a|x) is the conditional probability density

(A3) $$P\left(a\;|\;x\right)\;=\;\frac{P(x,a)}{P(x)}$$

and P(x) is the marginal of *x*

(A4) P(x)=∫P(x,a)da

Multiplying Equation (A2) by P(x) and integrating both sides with respect to *x*, we have

(A5) $$\int {\langle a\rangle }_{x}P\left(x\right)dx=\int aP\left(x,a\right)dadx$$

which shows that the global averages may be obtained by integrating the conditional average with the probability of *x*.

(A6) $$\langle a\rangle =\int {\langle a\rangle }_{x}P\left(x\right)dx$$

Now consider the relation between the global standard deviation, $\sigma_{a}^{2},$

(A7) $$\sigma_{a}^{2}=\langle \;\;a^{2}\;\;\rangle -{\langle a\rangle }^{2}$$

and the conditional standard deviation, $\sigma_{a|x}^{2}$, given by

(A8) $$\sigma_{a|x}^{2}={\langle a^{2}\rangle }_{x}-{\langle a\rangle }_{x}^{2}$$

Starting with the definition of $\sigma_{a|x}^{2}$

$$\begin{array}{cc} \sigma_{a|x}^{2} & =\int {\left(a-{\langle a\rangle }_{x}\right)}^{2}P\left(a\;|\;x\right)da\\ \; & =\frac{1}{P(x)}\int {\left(a-{\langle a\rangle }_{x}\right)}^{2}P(x,a)da \end{array}$$

multiply both sides by P(x) and integrate to obtain

(A9) $$\begin{array}{cc} \int \sigma_{a|x}^{2}\;P\left(x\right)\;dx\; & =\;\;\int \int \;{\left(\;a-{\langle \;a\;\rangle }_{x}\;\right)}^{2}\;P(x,a)\;da\;dx \end{array}$$

(A10) $$\begin{array}{cc} \; & \;=\;\int \int \;\left(\;a^{2}-2a{\langle \;a\;\rangle }_{x}+{\langle \;a\;\rangle }_{x}^{2}\;\right)\;P(x,a)\;da\;dx \end{array}$$

(A11) $$\begin{array}{cc} \; & \;=\;\;\langle a^{2}\rangle \;-\;\int {\langle \;a\;\rangle }_{x}^{2}\;P\left(x\right)\;dx \end{array}$$

The first term of the right-hand side gives $\;\langle a^{2}\rangle$, and for the second term, the integration gives

(A12) $$\int \int a{\langle \;a\;\rangle }_{x}P(x,a)\;da\;dx=\int \int {\langle \;a\;\rangle }_{x}{\langle a\rangle }_{x}P\left(x\right)\;dx$$

where we have used

(A13) $$\int a\;\;P(x,a)\;da={\langle a\rangle }_{x}P\left(x\right)$$

Hence,

(A14) $$\int \sigma_{a|x}^{2}\;P\left(x\right)\;dx\;=\;\;\langle \;a^{2}\;\rangle \;-\;\int {\langle \;a\;\rangle }_{x}^{2}\;P\left(x\right)\;dx$$

Now subtract and add ${\langle \;a\;\rangle }^{2}$ to the right-hand side of Equation (A14) to obtain

(A15) $$\begin{array}{cc} \int \sigma_{a|x}^{2}\;P\left(x\right)\;dx & =\sigma_{a}^{2}+{\langle \;a\;\rangle }^{2}-\int {\langle \;a\;\rangle }_{x}^{2}\;P\left(x\right)\;dx \end{array}$$

(A16) $$\begin{array}{cc} \; & \;=\sigma_{a}^{2}-\int {\left(\;{\langle \;a\;\rangle }_{x}-\langle \;a\;\;\rangle \;\right)}^{2}\;P\left(x\right)\;dx \end{array}$$

Therefore,

(A17) $$\sigma_{a}^{2}=\int \sigma_{a|x}^{2}\;P\left(x\right)\;db\;+\int {\left({\langle a\rangle }_{x}-\langle a\rangle \right)}^{2}\;P\left(x\right)\;dx$$

giving Equation (12) of the text. Note that in the above derivation, there is nothing special about *x*. It could be any other random variable.

### Appendix A.1. Quantum Uncertainty Equation

The quantum standard deviation in the position representation is given by

(A18) $${\left(\Delta A\right)}^{2}=\int \psi^{*}\left(x\right){\left(A-\langle A\rangle \right)}^{2}\psi \left(x\right)dx$$

Since the operator is Hermitian, we can write

(A19) $$\begin{array}{cc} {\left(\Delta A\right)}^{2} & =\int {\left|\left(\;\;A-\langle A\rangle \right)\psi \left(x\right)\right|}^{2}dx \end{array}$$

(A20) $$\begin{array}{cc} \; & \;=\int {\left|\left(\;\;\frac{A\psi (x)}{\psi (x)}-\langle A\rangle \right)\psi \left(x\right)\right|}^{2}dx \end{array}$$

Now break up

(A21) $$\;\;\frac{A\psi (x)}{\psi (x)}={\left(\frac{A\psi }{\psi }\right)}_{R}+i{\left(\frac{A\psi }{\psi }\right)}_{I}$$

and write

(A22) $${\left(\Delta A\right)}^{2}=\int {\left|\left[\;\;{\left(\frac{A\psi }{\psi }\right)}_{R}-\langle A\rangle +i{\left(\frac{A\psi }{\psi }\right)}_{I}\right]\psi \left(x\right)\right|}^{2}dx$$

The first two terms are real, and hence, we have that

(A23) $${\left(\Delta A\right)}^{2}=\int {\left(\frac{A\psi }{\psi }\right)}_{I}^{2}{\;|\psi \left(x\right)|}^{2}\;dx\;+\;\int {\left[\;{\left(\frac{A\psi }{\psi }\right)}_{R}\;\;-\langle A\rangle \;\right]}^{2}{\left|\psi \left(x\right)\right|}^{2}\;dx$$

(A24) $${\left(\Delta A\right)}^{2}=\int {\left(\frac{A\psi }{\psi }\right)}_{I}^{2}{\;|\psi \left(x\right)|}^{2}\;dx\;+\;\int \left[\;{\left(\frac{A\psi }{\psi }\right)}_{R}^{2}\;-\;{\langle A\rangle }^{2}\;\right]{\left|\psi \left(x\right)\right|}^{2}\;dx\;$$

### Appendix A.2. Momentum Representation

If we are in the momentum representation, the identical derivation gives

(A25) $${\left(\Delta A\right)}^{2}=\int {\left(\frac{A\varphi }{\varphi }\right)}_{I}^{2}{\;|\varphi \left(p\right)|}^{2}\;dp\;+\;\int {\left[\;{\left(\frac{A\varphi }{\varphi }\right)}_{R}\;-\;\langle A\rangle \;\right]}^{2}{\left|\varphi \left(p\right)\right|}^{2}\;dp\;$$

where φ(p) is the momentum wave function and the operator A has to be expressed in the momentum representation.

### Appendix A.3. Two Arbitrary Operators

For a Hermitian operator B, we write the eigenvalue problem

(A26) $$Bu_{\beta }\left(x\right)=\beta u_{\beta }\left(x\right)$$

where β and $u_{\beta }\left(x\right)$ are the eigenvalues and eigenfunctions, respectively. The wave function can be expanded as

(A27) $$\psi \left(x\right)=\int \eta \left(\beta \right)u_{\beta }\left(x\right)d\beta$$

with

(A28) $$\eta \left(\beta \right)=\int \psi \left(x\right)u_{\beta }^{*}\left(x\right)dx$$

where η(β) is the wave function in the beta representation. The expected value of an operator A expressed in the beta representation is

(A29) $$\langle A\rangle =\langle \eta \left|A\right|\eta \rangle =\int \eta^{*}\left(\beta \right)A\eta \left(\beta \right)d\beta$$

and the standard deviation is

(A30) $${\left(\Delta A\right)}^{2}=\langle \eta \left|A^{2}\right|\eta \rangle -{\langle \eta \left|A\right|\eta \rangle }^{2}=\int \eta^{*}\left(\beta \right){\left(A-\langle \eta \left|A\right|\eta \rangle \right)}^{2}\eta \left(\beta \right)d\beta$$

Again, we break up $\frac{A\eta }{\eta }$ into its real and imaginary parts

(A31) $$\frac{A\eta }{\eta }={\left(\frac{A\eta }{\eta }\right)}_{R}\;+\;i{\left(\frac{A\eta }{\eta }\right)}_{I}\;$$

Then, following the same steps as above, we have that the quantum mechanical standard deviation may be written as

(A32) $${\left(\Delta A\right)}^{2}=\int {\left(\frac{A\eta }{\eta }\right)}_{I}^{2}{\;|\eta \left(\beta \right)|}^{2}\;d\beta \;+\;\int {\left[\;{\left(\frac{A\eta }{\eta }\right)}_{R}\;-\;\langle A\rangle \;\right]}^{2}{\left|\eta \left(\beta \right)\right|}^{2}\;d\beta \;$$
